# Supplementary material for: Discovery of New Glucose Uptake Inhibitors as Potential Anticancer Agents by Non-Radioactive Cell-Based Assays
Source: Molecules. 2022 Nov 21;27(22):8106. doi: 10.3390/molecules27228106 (PMC9692963; doi:10.3390/molecules27228106)
Supplement: Supplementary file 1 [file molecules-27-08106-s001.zip › molecules-1983646-supplementary.pdf]

Phloretin

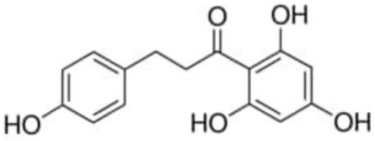

Resveratrol

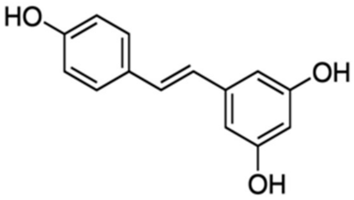

cytochalasin B

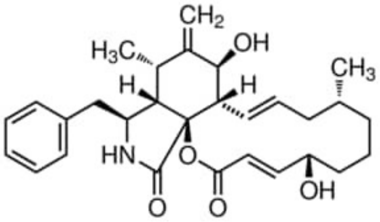

WZB117

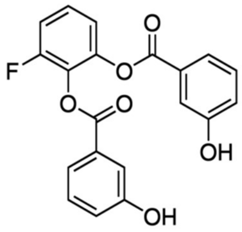

BAY-876

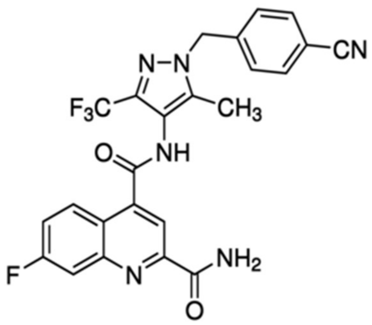

Figure S1. Chemical structure of known GLUT1 inhibitors phloretin, resveratrol, cytochalasin B, WZB117 and BAY-876.

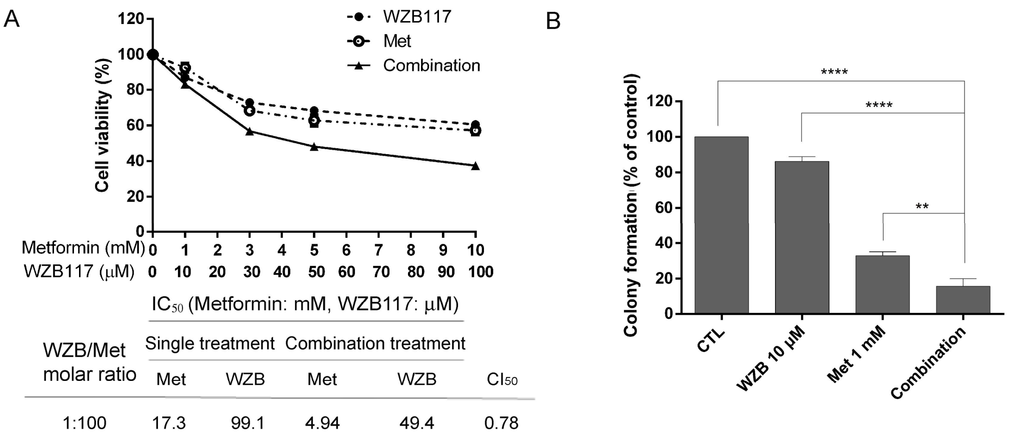

WZB/Met molar ratio	IC₅₀ (Metformin: mM, WZB117: μM)				
	Single treatment		Combination treatment		
	Met	WZB	Met	WZB	CI_{50}
1:100	17.3	99.1	4.94	49.4	0.78

Figure S2. WZB117 and metformin exhibit a synergistic anticancer effect in SKOV3 cells. (**A**) WZB117 synergized with metformin. Cells were seeded in 96-well plates and cell viability was measured by the MTT assay after 72 h of drug treatment. (**B**) Colony formation assay of the combination of WZB117 and metformin. Cells were seeded in 6-well plates and treated with indicated drugs for 10 days. CTL: vehicle control; Met: metformin; WZB: WZB117. Data are presented as mean ± SEM of at least three independent experiments. Statistical significance was assessed by one-way ANOVA. **, $P < 0.01$; ****, $P < 0.0001$.

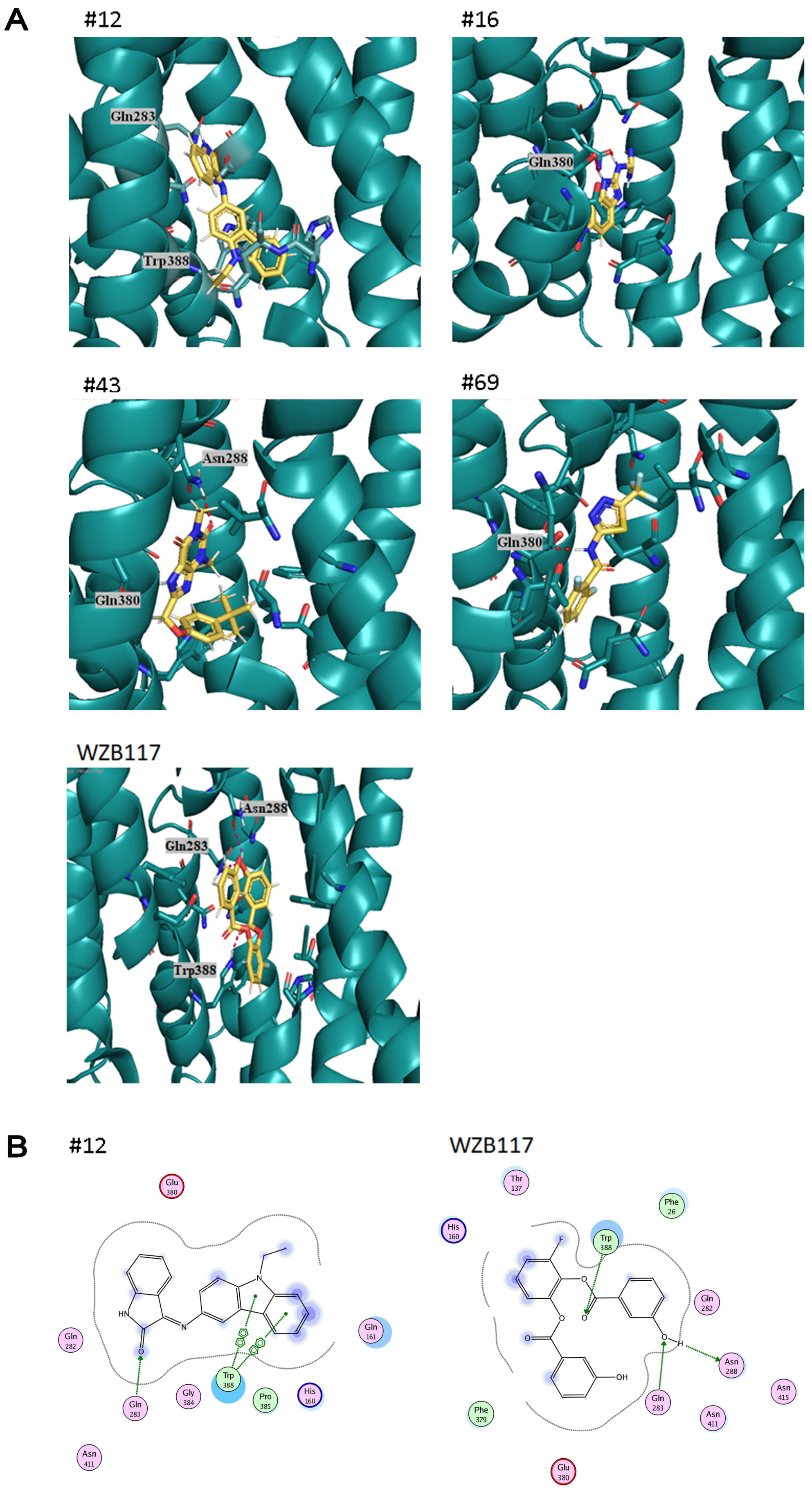
A
#12
Gln283
Trp388
#16
Gln380
#43
Asn288
Gln380
#69
Gln380
WZB117
Asn288
Gln283
Trp388
B
#12
Glu 380
Gln 282
Gln 161
Trp 388
Gln 283
Gly 384
Pro 385
His 160
Asn 411
HN
N
WZB117
Thr 137
Phe 26
His 160
Trp 388
Gln 282
Asn 288
Asn 415
Asn 411
Gln 283
Phe 379
Glu 380
F
O
OH
H

Figure S3. Molecular docking of GLUT1 inhibitors #12, #16, #43, #69 and WZB117. (**A**) Molecular docking analysis of new GLUT1 inhibitors #12, #16, #43 and #69, and WZB117 with hGLUT1. Hydrogen bonds are denoted with broken red lines. (**B**) Both #12 and WZB117 interacted with Gln283 and Trp388 of hGLUT1 via hydrogen bonds. X-ray structure of the hGLUT1 co-crystallized with a ligand n-nonyl-β-D-glucopyranoside (β-NG) in the IOP conformation (PDB ID: 4PYP) was retrieved from the RCSB Protein Data Bank. MOE software was used for the molecular docking analysis. PyMOL was then used to prepare the figures.

Table S1. The GLUT1 inhibitory activity of NCI compounds #1-#75 determined by high-throughput 2-NBDG uptake assay in COS-7 cells.

**Compound** [a]	**Uptake (%)**	**Compound** [a]	**Uptake (%)**
CTL	100	37	106.8 ± 6.97
Phloretin (50 μM)	56.38 ± 3.63	38	115.1 ± 9.91
Phloretin (100 μM)	53.83 ± 0.87	39	95.10 ± 4.30
1	89.58 ± 3.52	**40**	**79.49 ± 4.92**
2	91.11 ± 8.61	41	90.39 ± 4.57
3	*59.36 ± 5.38	42	91.75 ± 1.66
4	89.10 ± 3.18	**43**	**59.68 ± 2.28**
5	97.44 ± 4.43	44	108.5 ± 10.50
6	112.5 ± 6.30	45	107.3 ± 10.68
7	90.79 ± 3.91	**46**	**84.7 ± 5.81**
8	*22.37 ± 6.07	47	97.92 ± 5.41
9	87.38 ± 9.87	**48**	**82.52 ± 3.00**
10	93.33 ± 8.93	49	106.4 ± 7.37
**11**	**82.23 ± 5.51**	50	100.6 ± 6.48
**12**	**77.46 ± 3.82**	51	101.2 ± 6.51
13	93.93 ± 8.50	52	126.7 ± 5.50
**14**	**75.10 ± 8.84**	53	94.81 ± 5.88
15	87.44 ± 2.04	54	88.48 ± 4.07
**16**	**74.36 ± 2.99**	55	89.09 ± 5.51
17	115.4 ± 9.17	56	132.0 ± 15.97
18	97.22 ± 4.65	57	97.97 ± 1.39
19	*302.4 ± 11.46*	58	86.81 ± 8.14
20	99.59 ± 3.06	59	106.2 ± 8.21
21	87.54± 6.06	60	101.6 ± 3.94
22	102.2 ± 7.53	61	104.5 ± 5.09
23	93.37 ± 4.09	62	102.1 ± 7.48
24	96.93 ± 3.59	63	105.7 ± 9.6
25	*54.23 ± 3.54	64	103.4 ± 2.79
26	92.34 ± 4.81	65	88.40 ± 8.60
27	146.5 ± 13.9	66	111.1 ± 5.6
28	109.0 ± 9.81	67	98.4 ± 4.88
29	107.7 ± 6.58	68	91.43 ± 3.65
30	88.73 ± 8.64	**69**	**76.96 ± 5.91**
**31**	**79.83 ± 5.10**	70	119.4 ± 7.76

32	92.53 ± 5.23	71	86.89 ± 2.37
33	111.3 ± 3.20	*72*	*4900 ± 1013*
34	102.26 ± 8.11	73	109.5 ± 9.75
35	153.4 ± 10.1	74	105.8 ± 14.3
36	87.62 ± 4.09	75	91.47 ± 7.14

Data are presented as mean ± SEM of at least three independent experiments.

[a] The concentration of the test compounds was 100 μM (except #7, #18, #19, #28, #29: 20 μM; #60: 44 μM; #13, #14, #24, #31, #51, #66: 50 μM). The top 10 compounds highlighted in bold were further tested in SKOV3 cells. Two compounds with extremely high 2-NBDG uptake are highlighted in italic.

* indicated that the compounds caused severe cell loss.

Table S2. PCR primers.

Gene	Primer sequences	Product size*
hGLUT1	5’-TTGCAGGCTTCTCCAACTGGAC-3’<br>5’-CAGAACCAGGAGCAC AGTGAAG-3’	113 bp
hGLUT2	5'-AGCTGCGAATAAACAGGCAG-3'<br>5'-AAACTGGAAGGAACCCAGCA-3'	159 bp
hGAPDH	5’-GAAGGTGAAGGTCGGAGT-3’<br>5’-GAAGATGGTGATGGGATT-3’	226 bp

*The PCR conditions for *hGLUT1* and *hGAPDH* were initial denaturation at 95 ºC for 3 min, 35 (*hGLUT1*) or 40 (*hGAPDH*) cycles of denaturation at 95 ºC for 30 s, annealing at 55 ºC for 30 s, and extension at 72 ºC for 30 s, followed by a final extension at 72 ºC for 5 min. The PCR conditions for *hGLUT2* were initial denaturation at 95 ºC for 3 min, 40 cycles of denaturation at 95 ºC for 30 s, annealing and extension at 60 ºC for 60 s, followed by a final extension at 72 ºC for 5 min.
